# On the State of Public Health

**Published:** 1922-09

**Authors:** 


### "ON THE STATE OF PUBLIC HEALTH."

THE annual report of Sir George Newman, Chief Medical Officer of the Ministry of Health, is always an informative document, and is more readable than the ordinary official report. This year the report is of special interest in view of the continuous attacks made on the Ministry, on the one side by such persons as Lady Askwith, who objects to, and perpetually misrepresents, our national expenditure on public health, and, on the other side, by Mr. George Lansbury, who as inaccurately declares that the Ministry is now killing, instead of curing.

This report, published by H.M. Stationery Office at 3s., provides the necessary balance to these two extreme schools of thought. It answers the questions of the "Anti-Wasters," "What is the use of all our expenditure on health ?" by pointing out that millions of persons are not alive to-day, who died last year in Russia and elsewhere because there was no sanitary rate available to save them. "Public expenditure on national health is like expenditure on a lifeboat or a fire engine ; even more, it is like a long-term investment. . . . It is money hidden in maternity, in good schools, in pure food, in clean streets, in sanitary houses, in an abundant water supply, in dispensaries, hospitals, and sanatoria, and in the vast network of a sanitary and protective cordon. . . . Yet without this investment the nation is bankrupt." To those who argue that the Ministry's recent economies are killing, not curing, it points out that even the Geddes Committee recognised the necessity of efficiency of the Public Medical Services. In fact, in the current year more money will be devoted to essential health service than was contemplated by the committee as a result of drastic economies in other directions.

The report contains a number of new features, some of which have escaped the notice of the daily Press, which made much of the facts that last year England had the lowest death-rate ever recorded, and that recently there has been an improvement in health in the middle years of life, which were economically of the most value. The analysis of the percentages of the total amount of rates in England and Wales is of value to students of public health :—

| | Per cent. of total rates. | |
|---|---|---|
| Education (including public libraries) .. .. | 20·4 | |
| Highways (maintenance, repair, improvement, and scavenging, but not lighting) .. .. .. | 17·0 | |
| Health— | | |
| (i) Sewers ; removal, &c., of house refuse ; water supply ; parks ; baths ; cemeteries ; isolation hospitals ; vaccination .. .. .. .. .. | 17·1 | |
| (ii) Maternity and child welfare ; tuberculosis ; venereal diseases .. .. | 1·1 | |
| (iii) Lunacy and mental deficiency .. | 3·7 | |
| | | 21·9 |
| Housing .. .. .. .. .. .. | | 0·4 |
| Relief of the poor (excluding maintenance of lunatics in county and borough asylums) .. .. | | 13·7 |
| Administration of justice ; police ; fire brigades .. | | 7·9 |
| Other specific services .. .. .. .. | | 12·4 |
| Items common to two or more of the above-mentioned services, but not allocated to them in the Returns (general administrative expenses, &c.) | | 6·3 |
| Total .. .. .. .. .. .. | | 100·0 |

Critics often fail to realise that so small a percentage is spent on health services.

The section devoted to an examination of the results of National Insurance also contains much new and valuable material. It proves in a brilliant essay that the nation loses through sickness every year in England and Wales, among the insured population alone, the equivalent of the work of 278,000 persons, and also the labour and expense involved in their care during 14,476,000 weeks of their incapacitation. To estimate the national loss we have to add the time lost through sickness of the remaining 23,000,000 persons. The causes which lead to this grave amount of lost time have been analysed by means of the data provided in the medical record cards. The following short table represents months of expert work, and is, in fact, an inventory of the state of public health, indicating that minor maladies in the main are responsible for a mass of suffering and enforced idleness.

### Causes of Mortality.

The principal causes of mortality in England and Wales have been, as in former years :—

| | |
|---|---|
| Diseases of the heart .. .. | 53,707 |
| Diseases of the nervous system .. | 48,217 |
| Cancer .. .. .. .. | 46,022 |
| Tuberculosis .. .. .. | 42,678 |
| Pneumonia .. .. .. .. | 34,708 |
| Bronchitis .. .. .. .. | 33,684 |

Whooping-cough (4,576) was slightly more fatal, while measles (2,241) and diphtheria (4,772) caused many fewer deaths in 1921 than in 1920. Scarlet fever was responsible for 1,305 deaths, 125 less than in 1920. Small-pox caused five deaths, as compared

*Insured Persons in Representative Cities—Proportion of Certain Diseases to Total Cases.*—1916.

| Disease. | Total. No. | Total. Per 1,000 of Total. | Male. No. | Male. Per 1,000 of Total. | Female. No. | Female. Per 1,000 of Total |
|---|---|---|---|---|---|---|
| 1. Influenza .. .. | 3,097 | 85·3 | 2,004 | 90·2 | 1,093 | 77·5 |
| 2. Tuberculosis, all forms | 505 | 13·9 | 320 | 14·4 | 185 | 13·1 |
| 3. Organic heart disease | 520 | 14·3 | 355 | 16·0 | 165 | 11·7 |
| 4. Anæmia .. .. | 1,157 | 31·9 | 74 | 3·3 | 1,083 | 76·8 |
| 5. Bronchitis, bronchial and nasal catarrh, cold, &c. | 7,739 | 213·0 | 4,988 | 224·4 | 2,751 | 195·2 |
| 6. Pneumonia and other diseases of the respiratory system | 537 | 14·8 | 395 | 17·8 | 142 | 10·1 |
| 7. Diseases of digestive system | 4,766 | 131·2 | 2,797 | 125·8 | 1,969 | 139·6 |
| 8. Diseases of genito-urinary system | 912 | 25·1 | 339 | 15·3 | 573 | 40·6 |
| 9. Diseases of nervous system and special senses | 1,775 | 48·9 | 1,088 | 49·0 | 687 | 48·7 |
| 10. Skin diseases .. .. | 1,741 | 47·9 | 1,039 | 46·7 | 702 | 49·8 |
| 11. Injuries and accidents | 2,871 | 79·0 | 2,358 | 106·1 | 513 | 36·4 |
| 12. Abscess, boils, and other septic conditions | 2,503 | 68·9 | 1,647 | 74·1 | 856 | 60·7 |
| 13. Lumbago, rheumatism, &c. | 3,181 | 87·6 | 2,201 | 99·0 | 980 | 69·5 |
| 14. Debility, neuralgia and headache | 1,999 | 55·0 | 855 | 38·5 | 1,144 | 81·1 |
| 15. Malignant disease .. | 66 | 1·8 | 45 | 2·0 | 21 | 1·5 |
| 16. Other diseases .. | 2,956 | 81·4 | 1,720 | 77·4 | 1,236 | 87·7 |
| Total .. .. | 36,325 | 1000·0 | 22,225 | 1000·0 | 14,100 | 1000·0 |

with 30 in 1920. The number of cases was 336. The death rate for measles in 1921 was exceptionally low, but it is feared that in 1922 the mortality from this disease will show a substantial rise.

Of the total number of deaths registered during the year 70,250, or 15·3 per cent., were of infants under one year of age, yielding an infant mortality of 83, about the same rate as that of 1920. It is pointed out that the decline in infant mortality to 83 per 1,000 births in England and Wales in 1921 and to 88 per 1,000 births for the quinquennial period 1917-1921 are remarkable figures, and mean that (calculated on the average infant mortality of 1901-10) there was in 1921 a further saving of 38,000 infant lives. They also mean a better physical condition in children from one to five years of age, and a more enlightened understanding of private and public hygiene. There is, however, still room for further reduction before a reasonable standard has been reached. The closely related mortality among women in childbirth still remained high and had shown little or no improvement since 1894. No fewer than 3,323 women died in childbirth, and another 925 from conditions associated with it.

In order to reduce this excessive mortality and the mass of preventable sickness, Sir George Newman has common-sense remedies to suggest. The means are within the potentialities of to-day, for to initiate the necessary reforms neither new powers nor great expenditure are required. The matter is largely, almost wholly, one of education. It is by a multitude of small habits that the difference between a healthy and an unhealthy way of living is established, and it is the unhealthy way of living which gives victory, whether to the long-drawn-out, insidious endemic or the storming attack of epidemic disease. Yet neither by physique alone, nor by ventilation of work-places alone, will a security against disease be afforded. Temperance in food and drink is not alone a perfect defence. It is by the accumulation of all these safeguards, by temperance in food and drink, by the cultivation of good habits of body, by rational attention to the environment both of the work-place and the home, that an enduring defence against not one but all forms of disease is created."

